# Comparison of ultrasensitive and mass spectrometry quantification of blood-based amyloid biomarkers for Alzheimer’s disease diagnosis in a memory clinic cohort

**DOI:** 10.1186/s13195-023-01188-8

**Published:** 2023-02-18

**Authors:** Christophe Hirtz, Germain U. Busto, Karim Bennys, Jana Kindermans, Sophie Navucet, Laurent Tiers, Simone Lista, Jérôme Vialaret, Laure-Anne Gutierrez, Yves Dauvilliers, Claudine Berr, Sylvain Lehmann, Audrey Gabelle

**Affiliations:** 1 University of Montpellier, IRMB-PPC, INM, CHU Montpellier, INSERM CNRS, Montpellier, France; 2 Resource and Research Memory Center (CMRR), Department of Neurology, Montpellier University Hospital, 80 avenue Augustin Fliche, 34000 Montpellier, France; 3 Institute for Neurosciences of Montpellier (INM), Univ Montpellier, INSERM, Montpellier, France; 4 Sleep and Wake Disorders Center, Department of Neurology, Gui de Chauliac Hospital, University of Montpellier, Montpellier, France

**Keywords:** Alzheimer’s disease, Plasma, Biomarkers, IPMS, Simoa, Diagnosis

## Abstract

**Background:**

Alzheimer’s disease (AD) is a complex neurodegenerative disorder with β-amyloid pathology as a key underlying process. The relevance of cerebrospinal fluid (CSF) and brain imaging biomarkers is validated in clinical practice for early diagnosis. Yet, their cost and perceived invasiveness are a limitation for large-scale implementation. Based on positive amyloid profiles, blood-based biomarkers should allow to detect people at risk for AD and to monitor patients under therapeutics strategies. Thanks to the recent development of innovative proteomic tools, the sensibility and specificity of blood biomarkers have been considerably improved. However, their diagnosis and prognosis relevance for daily clinical practice is still incomplete.

**Methods:**

The Plasmaboost study included 184 participants from the Montpellier’s hospital NeuroCognition Biobank with AD (*n* = 73), mild cognitive impairments (MCI) (*n* = 32), subjective cognitive impairments (SCI) (*n* = 12), other neurodegenerative diseases (NDD) (*n* = 31), and other neurological disorders (OND) (*n* = 36). Dosage of β-amyloid biomarkers was performed on plasma samples using immunoprecipitation-mass spectrometry (IPMS) developed by Shimadzu (IPMS-Shim Aβ_42_, Aβ_40_, APP_669–711_) and Simoa Human Neurology 3-PLEX A assay (Aβ_42_, Aβ_40_, t-tau). Links between those biomarkers and demographical and clinical data and CSF AD biomarkers were investigated. Performances of the two technologies to discriminate clinically or biologically based (using the AT(N) framework) diagnosis of AD were compared using receiver operating characteristic (ROC) analyses.

**Results:**

The amyloid IPMS-Shim composite biomarker (combining APP_669–711_/Aβ_42_ and Aβ_40_/Aβ_42_ ratios) discriminated AD from SCI (AUC: 0.91), OND (0.89), and NDD (0.81). The IPMS-Shim Aβ_42/40_ ratio also discriminated AD from MCI (0.78). IPMS-Shim biomarkers have similar relevance to discriminate between amyloid-positive and amyloid-negative individuals (0.73 and 0.76 respectively) and A−T−N−/A+T+N+ profiles (0.83 and 0.85). Performances of the Simoa 3-PLEX Aβ_42/40_ ratio were more modest. Pilot longitudinal analysis on the progression of plasma biomarkers indicates that IPMS-Shim can detect the decrease in plasma Aβ_42_ that is specific to AD patients.

**Conclusions:**

Our study confirms the potential usefulness of amyloid plasma biomarkers, especially the IPMS-Shim technology, as a screening tool for early AD patients.

**Supplementary Information:**

The online version contains supplementary material available at 10.1186/s13195-023-01188-8.

## Background

Alzheimer’s disease (AD) is a complex, age-related neurodegenerative disorder, whose prevalence is anticipated to triple worldwide by 2050 [1]. With the introduction of molecular biomarkers, AD progressively acquired a biological definition that optimized the traditional clinical symptom-based approach [2]. Briefly, “A”, amyloid-beta (Aβ) plaques with amyloid precursor protein; “T”, neurofibrillary tangles of the hyperphosphorylated tau protein (p-tau); and “(N)”, neurodegeneration, taken together, define the AT(N) system, a biomarker-guided classification scheme categorizing individuals using the core pathophysiological features of the disease.

To date, for clinical routine, the quantification of biomarkers is based on the cerebrospinal fluid (CSF) concentration assessment of the 42-amino acid-long Aβ peptide (Aβ_42_) and/or the ratio between Aβ_42_ and the 40-amino acid-long Aβ peptide (Aβ_40_), hyperphosphorylated tau (p-tau), and total tau (t-tau) proteins [3] and/or neuroimaging techniques such as Aβ-positron emission tomography (PET) [4], tau-PET imaging [5], and structural magnetic resonance imaging (MRI) [6]. However, while highly performant, such tools require expensive imaging equipment, highly trained staff, and, for lumbar puncture, invasive procedures.

To overcome these constraints, blood-based biomarkers have been developed and results are promising, especially concerning the Aβ_42/40_ ratio; p-tau; neurofilament light chain (NfL), a marker of neuroaxonal injury; or glial fibrillary acidic protein (GFAP), a marker of glial activation [7–9]. Initial conflicting outcomes were later explained by the unavailability of immunoassays sensitive enough or possible misclassification of clinical diagnosis [8]. Recent advances both in mass spectrometry (MS) and immunodetection methods, together with standardization of preanalytical variables, allowed to partly overcome those limitations by improving sensitivity [10, 11].

Eight plasma Aβ_42/40_ assays were recently compared, in terms of performances, when detecting abnormal cerebral Aβ status (according to CSF Aβ_42/40_ or Aβ-PET imaging) in early AD patients [12]. Only two of them seem operable on a large scale but involve an arbitration between cost, flow, and performances: ultrasensitive single molecule array (Simoa) technology [13] or immunoprecipitation coupled with MS (IPMS), as developed by the Washington University or Shimadzu (IPMS-Shim) [14, 15].

A study relying on Simoa reported decreased plasma Aβ_40_ and Aβ_42_ concentrations and reduced Aβ_42/40_ in AD patients [16]. Such biomarkers could even discriminate mild cognitive impairment (MCI) from control individuals [16] and were relevant predictive tools of positive amyloid-PET status [17]. MS-based studies found similar results indicating that Aβ_42/40_ was inversely proportional to brain Aβ burden [15].

However, data are still incomplete in clinical practice and concerning the diagnostic and prognostic accuracy of Aβ plasma biomarkers to discriminate AD from MCI, individuals with subjective cognitive impairments (SCI), other neurodegenerative diseases (NDD), or other neurological disorders (OND). No comparative studies have been done between the most recent and relevant plasma biomarker dosages. In addition, it remains unclear whether plasma biomarkers have a better diagnostic usefulness based on core clinical or biological criteria (Aβ^−^/Aβ^+^, AT(N)). Eventually, the temporal changes in plasma amyloid biomarkers remained to be determined and explored using recent ultrasensitive proteomic technologies.

The main objective of our study was to determine, in a cohort of memory clinic patients with differential diagnosis and, for some of them, spread along the AD continuum, the diagnostic and prognosis relevance of the two most operable plasma amyloid biomarkers, ultrasensitive immunoassay and IPMS amyloid biomarker dosages.

## Methods

### Study participants

Participants were retrospectively selected from the NeuroCognition Biobank of Montpellier’s University Hospital including biological samples (plasma and CSF) from patients recruited in the Resource and Research Memory Center between 2007 and 2016. Patients gave informed and written consent to have their samples stored in an officially registered and ethically approved biological collection (#DC-2008-417) and later used for scientific research.

The diagnosis of AD patients was performed using the National Institute on Aging-Alzheimer’s Association (NIA-AA) criteria by Albert et al. [18] after multidisciplinary collegial meetings evaluating medical history, clinical symptoms, neuropsychological assessments, and neuroimaging (MRI), both prior and after the results of CSF Aβ_42_, t-tau, and p-tau_181_ biomarkers.

This allowed us to determine clinical AD diagnosis independently of the results of biomarkers, to dichotomize them into Aβ-positive (Aβ^+^) and Aβ-negative (Aβ^−^) participants, and to establish their AT(N) biological profiles [19]. Individuals established as AD patients with additional brain vascular lesions (*n* = 14) were diagnosed as mixed dementia [20] and combined with AD individuals to constitute the AD group. Twelve of them were amyloid-positive (CSF measurement, see below).

The MCI group was constituted based on Petersen’s criteria with memory or cognitive troubles without loss of autonomy [21]. Among MCI patients, nine were amyloid-positive, 19 negative, and four unknowns. The SCI group was defined as individuals with self-reported experience of worsening or more frequent confusion or memory loss, but without objective impairment in cognitive performance [22]. Details about the diagnosis included in the other neurodegenerative diseases (NDD) and other neurological disorders (OND) groups are available in Table S1.

### CSF biomarker measurement and cutoffs

CSF biomarkers were measured using standardized commercial Innotest sandwich ELISA (Fujirebio, *n* = 152) or Euroimmun ELISA method (*n* = 2) according to the manufacturer’s instructions. Sandwich ELISA relies on two antibodies for detection, one targeting the first 6 N-terminal amino acids (3D6) and the second the 6 C-terminal amino acids (21F12). The Aβ_42/40_ cutoff used to distinguish Aβ^+^ from Aβ^−^ individuals was 0.05, as preconized for clinical setting [23]. For the AT(N) research framework, participants were considered “A+” if CSF Aβ_42_ was < 500/700 pg/ml depending on the nature of the polypropylene collection tubes or if CSF Aβ_42/40_ ratio < 0.05/0.1 according to ELISA technic [24, 25]; “T+” if CSF p-tau_181_ was > 60 pg/ml; and N+ if CSF t-tau was > 400 pg/ml.

### Plasma biomarker samples

K2-EDTA blood samples were obtained through venipuncture. After a 10-min centrifugation at 1800×g within 2 h from collection, plasma was divided into 0.5-ml aliquots in 1.5–2-ml polypropylene tubes (Sarstedt, Germany) and stored at −80°C until biochemical assessment.

### Ultrasensitive Simoa immunoassay (Quanterix)

Samples were thawed at room temperature and centrifuged at 10,000×g for 10 min. Samples were measured using the commercial Simoa® Human Neurology 3-PLEX A assay (N3PA) (Quanterix). This assay relies on distinct antibodies to capture and to detect amyloid-β species (Aβ_42_, Aβ_40_). The capture antibody (6E10) recognizes the N-terminal region of both species (amino acids 4 to 10) while the detection antibodies are specific to the Aβ_42_ and Aβ_40_ C-terminal ends to reveal them [26]. Briefly, Aβ_42_, Aβ_40_, and t-tau were measured simultaneously in duplicates in 80-μl samples, according to the manufacturer’s instructions.

### IPMS-shim

The IPMS-Shim technology was slightly modified from Nakamura et al. [15]. Briefly, Aβ_42_, Aβ_40_, and APP_669–711_ were measured in 250-μl samples using a linear MALDI-TOF mass spectrometer (AXIMA Assurance, Shimadzu) after two consecutive IP steps with Dynabeads M-270 Epoxy used as beads and mouse monoclonal anti-Aβ antibodies to coat the beads. Aβ_42_ was then expressed relative to APP_669–711_ (also known as Aβ_3–40_) and Aβ_40_, both reflecting basal amyloid-β expression level. The IPMS-Shim composite biomarker was generated by combining the normalized score of APP_669–711_/Aβ_42_ and Aβ_40_/Aβ_42_ ratios, with Aβ_42_ as the denominator to obtain a normal distribution, as previously described [15].

### Statistical analysis

Data were analyzed using the R statistical software (version 4.0.2) [27]. For each group, quantitative variables were expressed as the median with the interquartile range (IQR, Q1 and Q3). Groups were compared using non-parametric Kruskal–Wallis or Mann–Whitney tests according to the group number. Pairwise comparisons were adjusted with Bonferroni correction. Correlations were assessed using Spearman’s rank correlation coefficient (*ρ*). The distribution of categorical variables was expressed with percentages and compared using Fisher’s exact test.

For comparisons of biomarker concentrations between diagnosis groups, to avoid the influence of extreme values, outliers were identified using Rosner’s test and discarded (*n* = 6). For each group, normal distribution was assessed using the Shapiro–Wilk test and homoscedasticity through Levene’s test. The assumption of normality was not obtained in only two groups, IPMS-Shim and Simoa Aβ_42/40_ for the AD diagnosis. ANCOVA’s assumptions of linearity, homogeneity of variance, non-collinearity of the factors (variance inflation factor <5), non-influential observations, and normality of residuals were evaluated. The impact of diagnosis on plasma amyloid biomarker concentrations was evaluated using ANCOVA controlling for age and *APOE* ε4 status. Multiple comparisons of the means were achieved using Tukey contrasts with diagnosis as a factor.

Plasma cutoffs were computed using expectation–maximization (EM) algorithms for mixtures of univariate normal distributions [28]. Cutoffs were visually determined at the intersection of two normal distributions.

A receiver operating characteristic (ROC) analysis was used to determine biomarker performances. A predictive formula adjusted for age and *APOE* ε4 status was built using a logistic regression analysis. The best values for sensitivity (se) and specificity (sp) were computed at an optimal cutoff point. Youden’s index was used to determine this optimal cutoff corresponding to the threshold maximizing the distance to the identity (diagonal) line and giving and equal weight to sensitivity and specificity. The area under the curve (AUC) was compared using the DeLong test. All tests were two-tailed, and significance was set at *α* = .05.

## Results

### Demographical, clinical, and CSF biomarker profiles

Characteristics of the 184 participants are summarized in Table 1. As expected, age was different between groups with participants in the OND group being significantly younger. *APOE* ε4 status also differed with a higher proportion of *APOE* ε4 carriers observed in AD patients relative to SCI and OND. Median MMSE scores were similar between AD, NDD, and OND groups, but differed, as expected, between AD, MCI, and SCI patients. The CSF biomarker profile supports the diagnosis of AD patients: CSF Aβ_42_ concentrations and Aβ_42/40_ ratio were reduced in the AD group relative to all other groups, while p-tau_181_ and t-tau concentrations were elevated compared to all other diagnoses.

**Table 1:** Plasmaboost cohort characteristics (*n* = 184)

Characteristic	Median (IQR)					***p*** value^**a**^		
	AD (***n*** = 73)	MCI (***n*** = 32)	SCI (***n*** = 12)	NDD (***n*** = 31)	OND (***n*** = 36)	AD vs. MCI/SCI	AD vs. NDD/OND	NAs
*APOE* ε4, No. (%)	24 (33)^b^	9 (28)	0 (0)	4 (13)	4 (11)	**0.045**	**0.014**	
Female, no. (%)	36 (49)	16 (50)	7 (58)	10 (32)	19 (53)	0.89	0.20	
Age, y	71 (68–76)	69 (64–77)	69 (62–72)	70 (66–77)	54 (35–67)^c^	0.50	**< 0.001**	
Education, y	9 (5–12)	9 (6–12)	14 (10–15)	9 (5–15)	11 (10–14)	0.20	0.47	3 | 1 | 3 | 1 | 26
MMSE, /30	24 (20–26)^d^	27 (26–29)	29 (27–30)	23 (19–28)	23 (17–27)	**< 0.001**	0.99	6 | 1 | 0 | 4 | 26
CSF, pg/ml								
Aβ_42_	578 (486–703)^e^	885 (609–1205)	800 (689–1477)	738 (590–1049)	971 (736–1206)	**< 0.001**	**< 0.001**	3 | 4 | 4 | 0 | 19
Aβ_42/40_	0.034 (0.027–0.045)^e^	0.065 (0.047–0.078)	0.062 (0.051–0 .072)	0.083 (0.055–0.096)	0.086 (0.048–0.097)	**< 0.001**	**< 0.001**	33 | 9 | 7 | 12 | 22
p-tau_181_	82 (66–106)^e^	45 (34–60)	41 (39–42)	33 (26–53)	33 (26–42)	**< 0.001**	**< 0.001**	3 |4 | 4 | 0 | 19
t-tau	630 (503–840)^e^	276 (229–391)	221 (200–228)	226 (170–388)	220 (144–340)	**< 0.001**	**< 0.001**	3 | 4 | 4 | 0 | 19

^a^Fisher’s exact (*APOE* ε4 and sex) or Kruskal–Wallis tests

^b^AD vs. MCI (*p* = 0.82), SCI (*p* = 0.017) or NDD (*p* = 0.052), OND (*p* = 0.019), Fisher’s exact test

^c^OND vs. AD (*p* < 0.001), NDD (*p* < 0.001), Mann–Whitney *U* tests with Bonferroni correction

^d^AD vs. MCI (*p* < 0.001), SCI (*p* < 0.001), Mann–Whitney *U* tests with Bonferroni correction

^e^AD vs. MCI (*p* < 0.05), SCI (*p* < 0.05) or NDD (*p* < 0.05), OND (*p* < 0.05), Mann–Whitney *U* tests with Bonferroni correction

We further explored potential associations between individual amyloid biomarkers and participants’ characteristics (age, education, MMSE, *APOE* ε4 status, and sex) to identify additional variables that might influence biomarker concentrations and potentially confound the impact of diagnosis (Table S2). The Aβ_42/40_ ratio, measured with Simoa and IPMS-Shim, decreased with age while the IPMS-Shim composite biomarker significantly increased with age. Same evolutions were observed for the *APOE* ε4 carriers. None of the other demographic characteristics (sex, education, and MMSE) was linked to plasma biomarker concentrations. At the scale of the cohort considered as a whole, plasma biomarkers are associated with age and *APOE* ε4 status, both were included in our ANCOVA and GLM models as potential confounding factors.

### Plasma amyloid biomarker characteristics

**Fig. 1:**
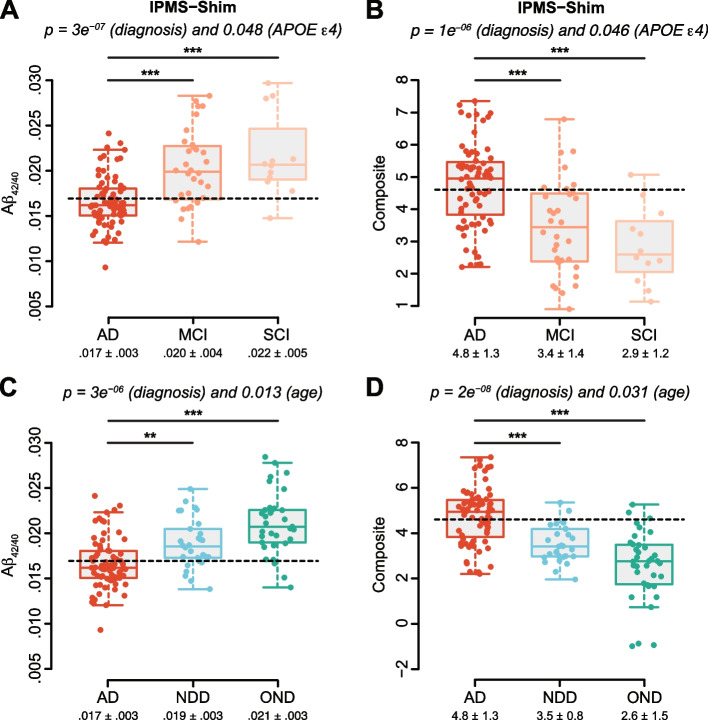
Levels of amyloid plasma biomarkers measured by IPMS and according to diagnosis. **A** Aβ_42/40_ ratio is decreased in the AD group relative to MCI and SCI with an effect of group and *APOE* ε4 status. **B** IPMS-Shim composite score is significantly greater in AD relative to both MCI and SCI groups with an effect of group and *APOE* ε4 status. **C** The Aβ_42/40_ ratio evaluated with IPMS-Shim is decreased in AD relative to NDD and OND groups with an effect of diagnosis and age. **D** The IPMS-Shim composite score is increased in AD relative to both groups with an effect of diagnosis and age. Plasma cutoffs, 0.017 for IPMS-Shim-Aβ_42/40_ and 4.6 for IPMS composite score, are represented with dashed lines. Results are boxplot, and mean ± SD are indicated below, *n* = 12–73. ANCOVA with diagnosis, age, and *APOE* ε4 status as factors followed by multiple comparisons of means using Tukey contrasts and diagnosis as a factor; ***p < 0.001, **p < 0.01

**Fig. 2:**
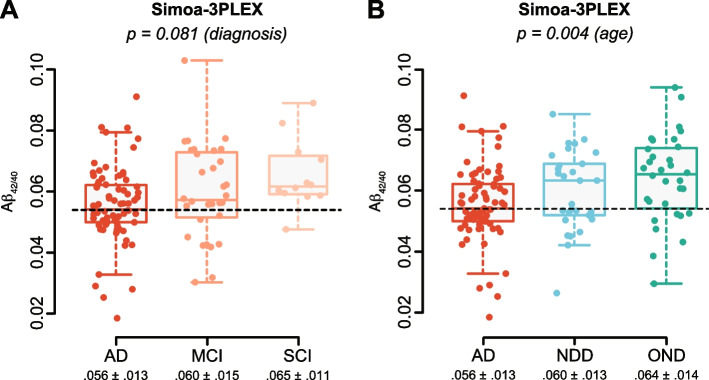
Levels of amyloid plasma biomarkers measured by Simoa-3PLEX and according to diagnosis. **A** Simoa Aβ_42/40_ ratio is not significantly different between diagnoses. **B** The Aβ_42/40_ ratio is globally different between groups when measured with Simoa but associated with an effect of age only. Plasma cutoff for Simoa-Aβ_42/40_, 0.054, is represented with dashed lines. Results are boxplot, and mean ± SD are indicated below, *n* = 12–73. ANCOVA with diagnosis, age, and *APOE* ε4 status as factors followed by multiple comparisons of means using Tukey contrasts and diagnosis as a factor

The IPMS-Shim Aβ_42/40_ ratio was significantly reduced in the AD group relative to both MCI and SCI groups (Fig. 1A) and similarly the IPMS-Shim composite score was increased (Fig. 1B). These two biomarkers were also able to distinguish AD from NDD and OND groups (Fig. 1C, D) with a combined effect of diagnosis and age for IPMS-Shim Aβ_42/40_ and the composite score. A cutoff to discriminate between AD and MCI/SCI and between AD and NDD/ODN could be established at 0.017 for IPMS-Shim Aβ_42/40_ ratio and at 4.6 for the composite score. Simoa Aβ_42/40_ ratio values were not statistically different between AD, MCI, and SCI (Fig. 2A). For Simoa Aβ_42/40_, only an effect of age reached statistical significance (Fig. 2B). Exclusion of patients with mixed dementia from the AD group did not change the effect of diagnosis. The impact on biomarker concentrations is thus robust and not sensitive to the presence of those samples.

### Diagnosis relevance

**Fig. 3:**
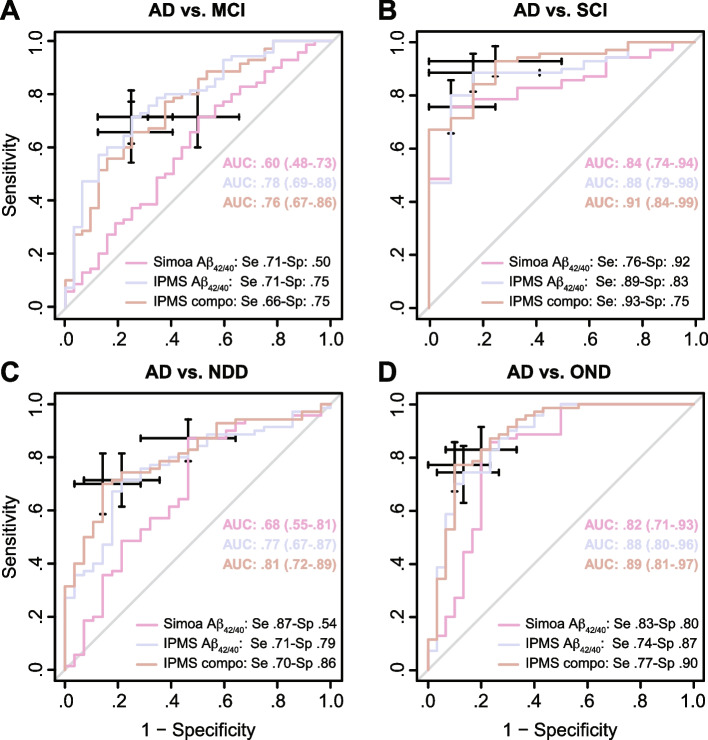
Performance of the plasma amyloid biomarkers to discriminate AD from other clinical diagnoses. **A** Receiver operating characteristic (ROC) analysis adjusted for age and *APOE* ε4 status of Simoa Aβ_42/40_, IPMS-Shim Aβ_42/40_, and composite biomarkers between AD and MCI groups. **B** ROC analysis of the three plasma amyloid biomarkers between AD and SCI groups. **C** ROC analysis of the biomarkers between AD and NDD groups. **D** ROC analysis of the biomarkers between AD and OND groups. AUC (area under the curve) is presented with 95% confidence interval (CI). The best values for sensitivity (se) and specificity (sp) were computed at an optimal cutoff point determined using Youden’s index

Diagnosis relevance of the biomarkers was computed for each diagnostic class individually, and models were adjusted for age and *APOE* ε4 status. Only the IPMS-Shim Aβ_42/40_ ratio and IPMS-Shim composite score were able to differentiate AD from MCI even with multivariate GLM adjustment, but not Simoa Aβ_42/40_ (Fig. 3A). IPMS-Shim Aβ_42/40_ had the largest AUC (0.78, 95% CI: 0.69–0.88) relative to the composite biomarker and both were significantly larger than Simoa Aβ_42/40_ (DeLong’s test; *p* < 0.01; Fig. 3A).

All three plasma biomarkers were able to discriminate AD from SCI in both univariate and multivariate models. While the IPMS-Shim composite score had the largest AUC (0.91, 0.84–0.99), it was not statistically different from the Aβ_42/40_ biomarkers (Fig. 3B).

To discriminate AD from NDD, the IPMS-Shim composite score had the largest adjusted AUC (0.81, 0.72–0.89) among the three biomarkers (Fig. 3C), even if not significantly different from Simoa Aβ_42/40_ and IPMS-Shim Aβ_42/40_. The IPMS-Shim Aβ_42/40_ ratio and IPMS-Shim composite score were both significant univariate and multivariate predictors of AD.

Simoa, IPMS-Shim Aβ_42/40_, and IPMS-Shim composite were able to discriminate AD from OND, but only IPMS-Shim Aβ_42/40_ and IPMS-Shim composite score remained significant after multivariate GLM adjustments (Fig. 3D). Taken together, the IPMS-Shim composite score had the largest adjusted AUC (0.89, 0.81–0.97). Simoa Aβ_42/40_ AUC was significantly smaller than the IPMS-Shim composite score (Fig. 3D).

### Consistency between plasma biomarkers and CSF Aβ_42/40_, AT(N) profile, and core clinical diagnosis

The CSF Aβ_42/40_ ratio was available for 103 individuals and the complete AT(N) profile for 112 among the 184 participants of the Plasmaboost cohort (Table S3). We assessed the ability of blood-based biomarkers to discriminate biologically confirmed cases using the dichotomy based on the CSF Aβ_42/40_ ratio and the AT(N) classification.

### Aβ^+^/Aβ^−^ dichotomy

**Fig. 4:**
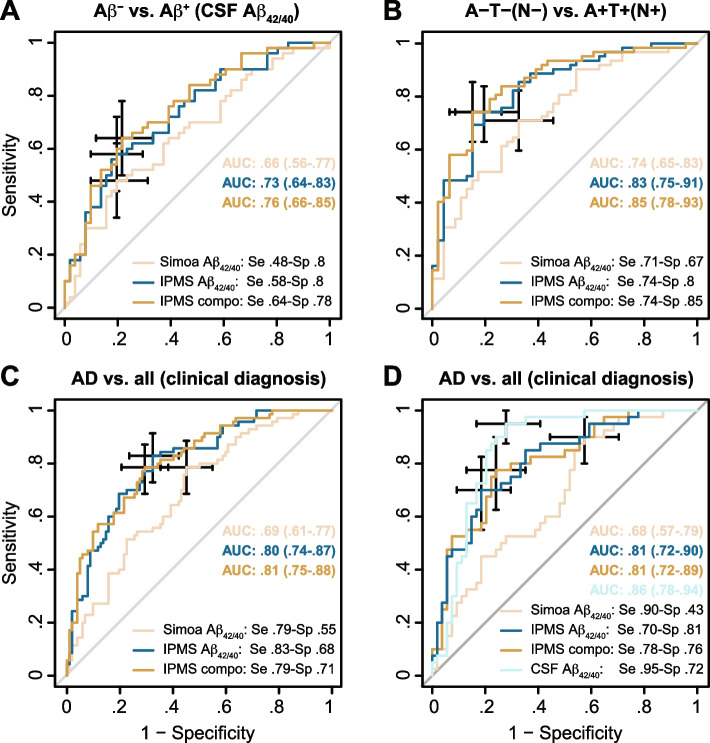
Performance of the plasma amyloid biomarkers to discriminate amyloid-positive (Aβ^+^) from amyloid-negative (Aβ^−^) subjects. **A** ROC analysis adjusted for age and *APOE* ε4 status of plasma Simoa Aβ_42/40_, IPMS-Shim Aβ_42/40_, and IPMS-Shim composite biomarkers between Aβ^+^ individuals and Aβ^−^. **B** ROC analysis of the amyloid biomarkers between A−T−N− and A+T+N+ participants. **C** ROC analysis of the three amyloid biomarkers between AD and all other diagnoses pooled together (non-AD). **D** ROC analysis of amyloid biomarkers between AD and other diagnoses for individuals with available CSF Aβ_42/40_ ratio. AUC is presented with 95% confidence interval (CI). The best values for sensitivity (se) and specificity (sp) were computed at an optimal cutoff point determined using Youden’s index

As expected, a greater proportion of AD patients belong to the Aβ^+^ group; however, the other characteristics were similar when comparing the Aβ^−^ and Aβ^+^ individuals (Table S3). The three plasma biomarkers were equivalent in discriminating Aβ^−^ from Aβ^+^ (Fig. 4A). AUC were between 0.66 and 0.76. Simoa Aβ_42/40_ was a significant predictor of Aβ^+^ status in a univariate model but the effect soothes to a statistical trend in a multivariate model (*p* = 0.06); this was not the case for the IPMS-Shim biomarkers which were both significant predictors in each type of model.

### AT(N) research framework

As for the Aβ group, there was a greater proportion of AD patients in the A+T+ and A+T+N+ groups (Table S3). Except for age, all other characteristics were similar between A−T− and A+T+ participants; A+T+N+ also had a greater proportion of *APOE* ε4 carriers (Table S3). All three plasma amyloid biomarkers significantly discriminate A−T− from A+T+ profiles even when models were adjusted for age and *APOE* ε4 status (Fig. S1). Both IPMS-Shim biomarkers have higher performances than Simoa Aβ_42/40_. All three biomarkers similarly discriminate A−T−N− from A+T+N+ profiles including after adjustment for age and *APOE* ε4 status (Fig. 4B). Interestingly, Simoa Aβ_42/40_ had significantly lower AUC than IPMS-Shim composite but similar to IPMS-Shim Aβ_42/40_.

To compare the discriminative ability of the plasma biomarkers using a biological or a clinical set-up, we pulled together all non-AD diagnoses (see Table 1 and Fig. 1). For all three plasma biomarkers, AUC ranged from 0.69 to 0.81 to discriminate AD against other diagnoses with the IPMS-Shim scores having significantly higher AUC than Simoa Aβ_42/40_ (Fig. 4C). Interestingly, Simoa was able to discriminate AD from other types of diagnosis even when adjusted for age and *APOE* ε4 status.

Eventually, to assess the performances of plasma biomarkers versus CSF, all participants with CSF Aβ_42/40_ and a diagnosis were compared (Fig. 4D). IPMS-Shim plasma biomarkers and CSF Aβ_42/40_ showed comparable discriminative power but CSF Aβ_42/40_ performance remained better than Simoa Aβ_42/40_.

### Correlations between CSF and plasma biomarkers

We compared technological platforms using the Passing-Bablok regression fit [29]. Results indicated that IPMS and Simoa were not equivalent when quantifying Aβ_42_, Aβ_40_, and Aβ_42/40_ ratio, confirming the performances measured with AUC, sensitivity, and sensibility. We further explored correlations between Simoa and IPMS-Shim Aβ_42_, Aβ_40_, and Aβ_42/40_ ratio plasma measurements with CSF Aβ_42_, Aβ_40_, and Aβ_42/40_ ratio results (Table S4). IPMS-Shim Aβ_42_ and Simoa Aβ_42_ correlated, as Aβ_40_ and Aβ_42/40_ ratios. Only IPMS-Shim Aβ_42_ was weakly correlated with CSF Aβ_42_ but not Simoa Aβ_42_. However, IPMS-Shim Aβ_42/40_ was significantly correlated with CSF Aβ_42/40_ as Simoa Aβ_42/40_. Eventually, none of the plasma Aβ_40_ was correlated with CSF Aβ_40_.

### Evolution of plasma biomarkers through time

Plasma biomarker levels were measured a second time, after 2 years (± 352 days), in 29 participants (10 AD, 8 MCI, 5 SCI, 3 NDD, and 3 OND). Characteristics were equivalent between AD and non-AD (Table S5). Individuals from the AD group exhibited lower MMSE in relation with the cognitive decline expected in AD. None of the SCI or MCI subjects converted to AD. Among CSF biomarkers, only p-tau_181_ and t-tau were increased in the AD group; however, results should be taken with caution given the small number of subjects in this exploratory study. Interestingly, both IPMS-Shim plasma biomarkers were significantly altered in the AD group at baseline while this was not the case for Simoa Aβ_42/40_ ratio.

**Fig. 5:**
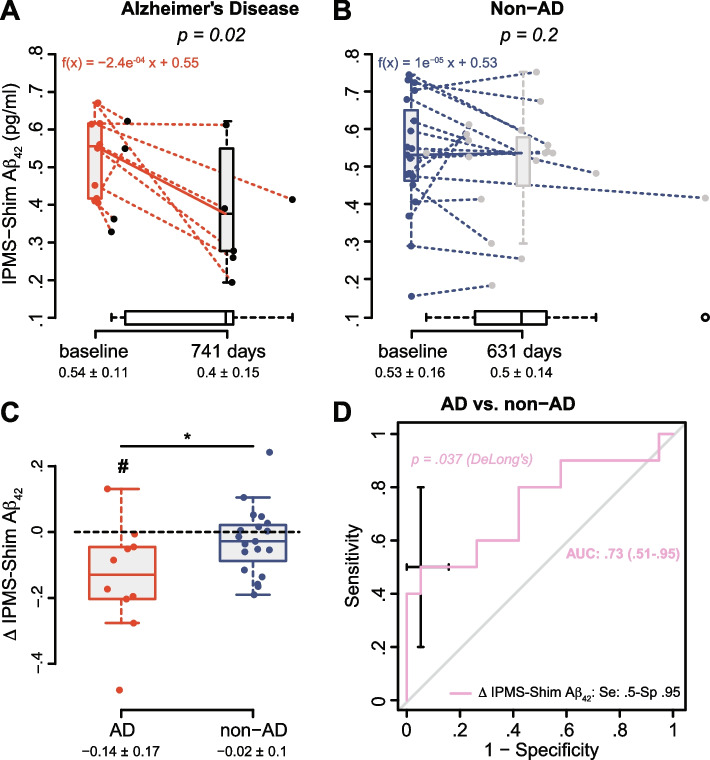
Evolution of the plasma IPMS-Shim Aβ_42_ biomarker through time and ability to discriminate AD subjects. **A** Variation in IPMS-Shim Aβ_42_ concentrations after a median of 741 (172–783) days in AD subjects. **B** Evolution in IPMS-Shim Aβ_42_ levels after a median of 631 (366–773) days in non-AD subjects. Results are boxplots with individual values at baseline and follow-up, and mean ± SD are indicated below with *n* = 10 and 19. Dotted lines connect the values of the same individual. Horizontal boxplots represent delay distribution. The decline was expressed as a linear function because only two time points were available. Wilcoxon signed-rank test for paired data. **C** Change (Δ) in IPMS-Shim Aβ_42_ for the AD and non-AD groups. One-sample Mann–Whitney test, ^#^*p* < 0.05; Mann–Whitney test, *p < 0.05. **D** ROC/AUC analysis of the change in IPMS-Shim Aβ_42_ between AD and non-AD groups. AUC is presented with 95% confidence interval (CI). The best values for sensitivity (se) and specificity (sp) were computed at an optimal cutoff point determined using Youden’s index

Among all plasma biomarkers tested, only IPMS Aβ_40_, Aβ_42_, and Simoa Aβ_42/40_ exhibited significant changes after ~2 years (Table 2). Interestingly, the decrease in IPMS-Shim Aβ_42_ was specific to the AD group. We thus further explored IPMS Aβ_42_ and showed a significant decrease after a follow-up of 741 days on average and with a mean rate of decline of 0.08 pg/ml/year (Fig. 5A). This was not the case for the non-AD group (Fig. 5B). We further plotted the variation in Aβ_42_ concentrations to show the average change over 2 years that was statistically different from 0 and from the non-AD group (Fig. 5C). We then estimated the ability of the decrease in IPMS-shim Aβ_42_ to predict AD status (GLM/ROC) and showed an AUC of 0.73 (Fig. 5D) which was statistically different from random and with a specificity of 0.95. Eventually, for the four participants with a change in IPMS-shim Aβ_42_ below −0.188, we showed that this decrease could be predicted (GLM) by a low MMSE at baseline (*p* = 0.03).

**Table 2:** Baseline and follow-up amyloid biomarker concentrations according to diagnosis and analytical technique

Biomarker	AD (***n*** = 10)		***p*** value^**a**^	Non-AD (***n*** = 19)		***p*** value^**a**^
	Baseline	Follow-up		Baseline	Follow-up	
Simoa Aβ_40_	233 (194–283)	198 (180–274)	0.94	230 (180–254)	197 (149–224)	0.18
IPMS-Shim Aβ_40_	36 (28–38)	23 (18–33)	**0.027**	29 (24–33)	27 (22–30)	**0.045**
Simoa Aβ_42_	11 (9–13)	10 (8–13)	0.94	13 (11–15)	12 (9–13)	0.71
IPMS-Shim Aβ_42_	0.55 (0.42–0.61)	0.37 (0.29–0.51)	**0.020**	0.53 (0.46–0.65)	0.54 (0.45–0.58)	0.23
Simoa Aβ_42/40_	0.055 (0.041–0.057)	0.054 (0.042–0.057)	0.69	0.057 (0.052–0.062)	0.058 (0.054–0.077)	**0.044**
IPMS-Shim Aβ_42/40_	0.016 (0.015–0.018)	0.016 (0.015–0.017)	0.56	0.018 (0.017–0.021)	0.02 (0.018–0.023)	0.17
IPMS-Shim composite	5.1 (4.7–5.3)	5.4 (4.5–6.2)	0.77	4 (2.7–4.7)	3.1 (2.1–3.9)	0.14

Biomarker concentrations are expressed as the median with (IQR, Q1–Q3) in pg/ml

*Abbreviations*: AD, Alzheimer’s disease; non-AD: 8 MCI, 5 SCI, 3 NDD, and 3 OND; Aβ_*40*_, 40-amino acid-long Aβ peptide; Aβ_*42*_ 42-amino acid-long Aβ peptide

^a^Wilcoxon signed-rank test (for paired data)

## Discussion

We validated, using samples obtained in a memory clinic, the diagnostic relevance of the IPMS-Shim composite score to discriminate clinical AD—in the early stages of the disease—from MCI, SCI, OND, and NDD (Fig. 3). IPMS-Shim plasma Aβ_42_ measurements and Aβ_42/40_ ratio were weakly but significantly correlated with CSF Aβ_42_ and Aβ_42/40_ results (Table S2). In contrast, Simoa 3-PLEX did not achieve IPMS-Shim diagnostic performances and failed to correlate with the core biomarkers, at least for Aβ_42_.

The positive correlation between plasma IPMS-Shim Aβ_42/40_ measurements and CSF values was replicated, as previously indicated [14]. Moreover, as described by Janelidze and colleagues, we confirmed the lack of correlation between CSF and plasma using the Simoa 3-PLEX technology [16]. It was later discovered that a substantial non-specific Aβ_3–42_ signal was measured using this assay due to the region targeted by the capture antibody (amino acid 4 to 10) [30]. Alternatively, quantification in the CSF employs the highly specific sandwich ELISA technique, potentially explaining the lack of correlation with the 3-PLEX and the modest performances of the 3-PLEX assay. Thijsenn et al. recently developed full-length antibodies against Aβ_40_ and Aβ_42_ that indeed revealed better sensitivity and specificity than the 3-PLEX [30] and used for the development of a new assay (4-PLEX).

IPMS-Shim-based biomarkers revealed better diagnostic performances in all clinical categories, which could be explained by the high specificity of MS-based technologies, in general [31], and the better performances compared with those of immunoassays [12]. Moreover, MS minimizes the matrix effect observed in the blood [32]. Eventually, multiple pathological conditions (inflammation, renal dysfunction…) alter or at least affect basal amyloid-β expression and might cause inter-individual variations, especially in the plasma. As shown by others, expressing Aβ_42_ relative to a reference, as APP_669–711_, improves its discriminative performance [33]. Expressing Aβ_42_ relative to two references, combined in a composite score, exhibit even higher performances [15].

We were able to reveal a decrease of IPMS-Shim Aβ_42_ that seemed to be specific to the AD diagnosis (Table S5 and Fig. 5A, B). Even if these results are exploratory and should be confirmed on a larger cohort, this is the first description, to our knowledge, of such evolution of plasma Aβ_42_ using ultrasensitive methods. An important change (< −0.188 over ~2 years) in plasma Aβ_42_ concentrations could reveal a useful biomarker to detect AD patients as it is highly specific of the disease (Sp = 0.95). The data available in the literature indicate a drop in plasma Aβ_42_ in the early phases of the disease in healthy controls transitioning to MCI [34] or MCI to AD [35], consistent with the decrease observed with CSF Aβ_42_ [36]. Additional studies incorporating multiple time points and using state-of-the-art technologies will be necessary to conclude on the evolution of Aβ_42_ in the plasma.

Aβ_42/40_ ratio was further explored since there is growing evidence emphasizing its role as a potentially better diagnostic biomarker than the absolute value of Aβ_42_, at least in CSF analyses [37]. Plasma Aβ_42/40_ was reduced in AD patients relative to NDD, OND, MCI, and SCI participants (Fig. 1). We confirmed the results found by other studies that used Simoa [16] and MS [15, 38, 39]. Moreover, when Aβ^+^, A+T+, or A+T+N+ individuals were investigated, this ratio was reduced using all strategies (Table S3). Taken together, those results emphasize the potential role of low plasma Aβ_42/40_ concentrations as a robust indicator of both AD clinical diagnosis and biologically confirmed cases.

Plasma p-tau is assumed to be another attractive blood-based candidate biomarker for AD clinical diagnosis. However, p-tau, which is stable in CSF, exhibits a very short half-life (around 10 hours) in blood [40] and may appear later during the progression of the disease [41]. Eventually, t-tau, considered as a biomarker of neuronal injury in the CSF but susceptible to degradation by proteases in the plasma, might be replaced, for an initial blood-based diagnostic, by NfL. NfL is a more promising biomarker, robust in the plasma, whose concentration increases with neurodegeneration, that would allow to identify patients at risk of cognitive decline and to track disease progression [8].

Our study presents some limits. First, the number of individuals in the SCI group and with available CSF Aβ_42/40_ concentrations was limited. This was also the case for the longitudinal analysis; however, given the specificity in Aβ_42_ decrease, it appeared worth reporting. Second, a few characteristics (MMSE, education, CSF biomarkers) were not available for the OND group because it did not require the same set of procedures as the other groups in a memory clinic.

One of the strengths of our analysis is that it was conducted on a sample that reflects the population that attends memory clinics in France. None of the highly selective inclusion or exclusion criteria generally used for clinical research was used. Our sample, while heterogeneous, thus mirror the diversity of AD presentations. The most up-to-date and operable proteomic techniques were used for biomarker quantification. Our results confirm that they could be implemented for AD pre-screenings in memory clinics before further expensive or invasive tests and with diagnosis performances similar to CSF measures.

## Conclusions

There is no doubt that the real diagnostic potential of plasma biomarkers will be achieved by developing molecular panels combining several of them [42]. Indeed, mounting evidence indicates that AD may present, even at the preclinical stage, a complex molecular signature; this can be deduced from peripheral blood analyses. Hence, using panels of blood biomarkers is supposed to outperform single candidate biomarkers in terms of AD diagnosis and prognosis [40, 43]. Unquestionably, blood (plasma)-based biomarkers are expected to play a crucial role in both AD diagnosis and prognosis, and in the therapeutic practice of the disease, in the upcoming future.

## Supplementary Information

**Additional file 1: Table S1.** Details about the diagnosis in the other neurodegenerative diseases (NDD) and other neurological disorders (OND) groups. **Table S2.** Correlations between plasma Biomarkers with Simoa and IPMS-Shim and Demographic Features. **Table S3.** Characteristics of Study Participants According to CSF Aβ_42/40_ Status and AT or AT(N) Profiles. **Table S4.** Correlations between Plasma Amyloid Biomarkers and CSF/Plasma Amyloid Biomarkers. **Table S5.** Baseline Characteristics of the 29 Individuals with follow-up and repeated biomarkers assessment. **Fig. S1.** Performance of the plasma amyloid biomarkers to discriminate A+T+ from A-T- subjects. ROC analysis of the amyloid biomarkers. AUC is presented with 95% confidence interval (CI).

## Data Availability

Plasmaboost de-identifed data are available to qualified researchers upon approved request to the corresponding author.

## Abbreviations

IQR: Interquartile range (Q1–Q3)
AD: Alzheimer’s disease
MCI: Mild cognitive impairment
SCI: Subjective cognitive impairment
NDD: Other neurodegenerative diseases
OND: Other neurological disorders
NA: Non-available
y: Years
*APOE* ε4: ε4 allele of the gene encoding for the apolipoprotein E
No.: Number
MMSE: Mini-Mental State Examination
CSF: Cerebrospinal fluid
Aβ_42_ (also known as Aβ_1–42_): 42-Amino acid-long Aβ peptide
Aβ_40_ (also known as Aβ_1–40_): 40-Amino acid-long Aβ peptide
p-tau_181_: Tau hyperphosphorylated at threonine 181
t-tau: Total tau
