# Supplementary material for: Comparison of ultrasensitive and mass spectrometry quantification of blood-based amyloid biomarkers for Alzheimer’s disease diagnosis in a memory clinic cohort
Source: Alzheimers Res Ther. 2023 Feb 18;15:34. doi: 10.1186/s13195-023-01188-8 (PMC9938625; doi:10.1186/s13195-023-01188-8)

**Supplementary data**

**Table S1** Details about the diagnosis in the other neurodegenerative diseases (NDD) and other neurological disorders (OND) groups

| **Group** | **Diagnosis** | **N** |
| --- | --- | --- |
| NDD | Amyotrophic lateral sclerosis (ALS) | 1 |
|  | Corticobasal degeneration (CBD) | 2 |
|  | Dementia with Lewy bodies (DLB) | 5 |
|  | Frontotemporal dementia (FTD) | 12 |
|  | Parkinson’s disease (PD) | 4 |
|  | Progressive nuclear palsy (PSP) | 4 |
|  | ND of unknown cause | 3 |
| OND | angiopathy | 1 |
|  | Chronic inflammatory demyelinating polyneuropathy (CIDP) | 1 |
|  | Chronic hydrocephalus in adults (CHA) | 2 |
|  | encephalitis | 2 |
|  | epilepsy | 5 |
|  | headache | 1 |
|  | intracranial pressure | 2 |
|  | leukoaraiosis | 1 |
|  | multiple sclerosis | 8 |
|  | neoplasia | 3 |
|  | normal pressure hydrocephalus | 3 |
|  | psychiatric disorder | 2 |
|  | sensory disturbances | 2 |
|  | stroke | 1 |
|  | peripheral neuropathy | 2 |

**Table S2** Correlations between plasma Biomarkers with Simoa and IPMS-Shim and Demographic Features

|  |  |  |  | ***APOE*** ε**4** | | |  | **Sex** | | |
| --- | --- | --- | --- | --- | --- | --- | --- | --- | --- | --- |
| **Biomarker** | **Age** | **Education** | **MMSE** | - | + | ***P* value** |  | m | f | ***P* value** |
| Simoa Aβ_42/40_ | **- 0.16 (0.029)** | 0.08 (0.36) | 0.09 (0.32) | 0.060 (0.052 - 0.070) | 0.055 (0.047 - 0.065) | **0.022** |  | 0.058 (0.050 - 0.067) | 0.059 (0.052 - 0.071) | 0.39 |
| IPMS-Shim Aβ_42/40_ | **- 0.22 (< 0.01)** | 0.01 (0.92) | 0.09 (0.27) | 0.019 (0.016 - 0.022) | 0.017 (0.015 - 0.019) | **< 0.01** |  | 0.018 (0.016 - 0.021) | 0.019 (0.016 - 0.023) | 0.70 |
| IPMS-Shim Composite | **0.23 (< 0.01)** | - 0.04 (0.59) | - 0.14 (0.10) | 3.6 (2.7 - 4.7) | 4.6 (3.3 - 5.4) | **< 0.01** |  | 3.8 (3 - 5) | 3.7 (2.4 - 5) | 0.72 |

For quantitative characteristics (age, education and MMSE), Spearman's rank correlation coefficient (ρ) with biomarkers and the associated *P*-values are indicated in parenthesis. For qualitative characteristics (*APOE* ε4 allele status and sex), biomarkers are expressed in pg/ml. Median and interquartile range (IQR, Q1 - Q3) for each biomarker were computed according to the qualitative data category and compared through Mann-Whitney *U* tests.

**Table S3** Characteristics of Study Participants According to CSF Aβ_42/40_ Status and AT or AT(N) Profiles

|  | **Median (IQR)** |  |  |  | **Median (IQR)** |  |  |  | **Median (IQR)** |  |  |  |
| --- | --- | --- | --- | --- | --- | --- | --- | --- | --- | --- | --- | --- |
| **Characteristic** | Aβ negative (n = 52)^a^ | Aβ positive (n = 51)^a^ | ***P* value^b^** | **NAs** | A-T- (n = 57)^c^ | A+T+ (n = 67)^c^ | ***P* value^b^** | **NAs** | A-T-N- (n = 48)^d^ | A+T+N+ (n = 64)^a^ | ***P* value^b^** | **NAs** |
| Diagnosis (AD \| MCI \| SCI \| NDD \| OND), No. | **7** \| 16 \| 5 \| 14 \| 10 | **34** \| 7 \| 0 \| 6 \| 4 | **< 0.001** |  | **2** \| 16 \| 7 \| 21 \| 11 | **55** \| 4 \| 0 \| 6 \| 2 | **< 0.001** |  | **1** \| 13 \| 7 \| 18 \| 9 | **53** \| 4 \| 0 \| 5 \| 2 | **< 0.001** |  |
| *APOE* ε4, No. (%) | 10 (19) | 16 (31) | 0.19 |  | 8 (14) | 20 (30) | 0.052 |  | 5 (10) | 20 (31) | **0.011** |  |
| Female, No. (%) | 21 (40.4) | 25 (49) | 0.43 |  | 25 (44) | 35 (52) | 0.37 |  | 21 (44) | 34 (53) | 0.35 |  |
| Age, y | 70 (63 - 73) | 70 (66 - 76) | 0.37 |  | 69 (61 - 73) | 71 (68 - 77) | **0.03** |  | 68 (62 - 73) | 71 (68 - 76) | **0.046** |  |
| Education, y | 10 (9 - 14) | 9 (5 - 13) | 0.34 | 7 \| 4 | 10 (5 - 14) | 9 (5 - 12) | 0.30 | 10 \| 5 | 10 (5 - 15) | 9 (5 - 12) | 0.27 | 8 \| 5 |
| MMSE, /30 | 26 (23 - 28) | 24 (20 - 27) | **0.044** | 5 \| 7 | 27 (24 - 28) | 23 (20 - 26) | **< 0.001** | 9 \| 7 | 27 (24 - 28) | 23 (19 - 26) | **< 0.001** | 7 \| 7 |
| CSF, pg/ml |  |  |  |  |  |  |  |  |  |  |  |  |
| Aβ_42/40_ | 0.080 (0.065 - 0.093) | 0.032 (0.026 - 0.039) | **< 0.001** |  | 0.084 (0.073 - 0.097) | 0.032 (0.026 - 0.034) | **< 0.001** | 17 \| 24 | 0.086 (0.073 - 0.096) | 0.031 (0.026 - 0.039) | **< 0.001** | 14 \| 24 |
| Aβ_42_ | 967 (735 - 1307) | 568 (473 - 698) | **< 0.001** |  | 1003 (764 - 1306) | 551 (465 - 657) | **< 0.001** |  | 947 (735 - 1301) | 554 (471 - 659) | **< 0.001** |  |
| p-tau_181_ | 35 (28 - 53) | 79 (65 - 100) | **< 0.001** |  | 34 (27 - 41) | 86 (72 - 109) | **< 0.001** |  | 34 (27 - 39) | 89 (74 - 110) | **< 0.001** |  |
| t-tau | 226 (175 - 330) | 552 (420 - 717) | **< 0.001** |  | 217 (174 - 271) | 656 (525 - 882) | **< 0.001** |  | 200 (170 - 240) | 665 (538 - 912) | **< 0.001** |  |
| Plasma, pg/ml |  |  |  |  |  |  |  |  |  |  |  |  |
| IPMS-Shim - Aβ_42/40_ | 0.020 (0.017 - 0.022) | 0.017 (0.015 - 0.019) | **< 0.001** |  | 0.021 (0.018 - 0.023) | 0.016 (0.015 - 0.018) | **< 0.001** |  | 0.020 (0.018 - 0.023) | 0.016 (0.015 - 0.018) | **< 0.001** |  |
| IPMS-Shim - Composite | 3.15 (2.41 - 3.95) | 4.52 (3.61 - 5.33) | **< 0.001** |  | 2.93 (2.30 - 3.78) | 4.93 (3.96 - 5.46) | **< 0.001** |  | 3.00 (2.39 - 3.87) | 4.96 (4.08 - 5.44) | **< 0.001** |  |
| Simoa - Aβ_42/40_ | 0.059 (0.052 - 0.067) | 0.053 (0.048 - 0.062) | **0.022** | 1 \| 1 | 0.063 (0.055 - 0.072) | 0.054 (0.049 - 0.062) | **< 0.001** | 2 \| 2 | 0.063 (0.054 - 0.071) | 0.054 (0.049 - 0.062) | **< 0.001** | 2 \| 2 |

^a^: Aβ positive status was defined as CSF Aβ_42/40_ ratio < 0.05 or 0.1 according to technic

^b^: between group differences were assessed using Fisher’s exact (diagnosis, *APOE* ε4 status and sex) or Mann-Whitney *U* tests

^c^: A+, amyloid-β positive if CSF Aβ_42_ < 500/700 pg/ml or, when available, CSF Aβ_42/40_ ratio < 0.05/0.1; T+, phospho-tau positive if CSF p-tau_181_ > 60 pg/ml

^d^: A+, amyloid-β positive if CSF Aβ_42_ < 500/700 pg/ml or, when available, CSF Aβ_42/40_ ratio < 0.05/0.1; T+, phospho-tau positive if CSF p-tau_181_ > 60 pg/ml; N+, neurodegeneration positive if CSF Tau > 400 pg/ml or, when available, Scheltens score is ≥ 2 before 75 years old or ≥ 3 after 75

**Table S4** Correlations between Plasma Amyloid Biomarkers and CSF/Plasma Amyloid Biomarkers

| **Plasma** | Simoa |  |  |
| --- | --- | --- | --- |
|  | **Aβ_42_** | **Aβ_40_** | **Aβ_42/40_** |
| IPMS-Shim | 0.5 (**< 0.001**) | 0.59 (**< 0.001**) | 0.33 (**< 0.001**) |
|  | **CSF** |  |  |
| **Plasma** | **Aβ_42_** | **Aβ_40_** | **Aβ_42/40_** |
| Simoa | 0.085 (0.31) | - 0.009 (0.93) | 0.31 (**< 0.01**) |
| IPMS-Shim | 0.21 (**< 0.01**) | 0.031 (0.76) | 0.35 (**< 0.001**) |

Correlations between biomarkers are expressed with Spearman’s rank correlation coefficient (ρ) and the associated p-values (*P*) shown in parentheses.

Abbreviations: Aβ_40_, 40-amino acid-long Aβ peptide; Aβ_42_, 42-amino acid-long Aβ peptide; CSF, cerebrospinal fluid.

**Table S5** Baseline Characteristics of the 29 Individuals with follow-up and repeated biomarkers assessment

|  | **Median (IQR)** |  |  |  |
| --- | --- | --- | --- | --- |
| **Characteristic** | AD (n = 10) | Non-AD^a^ (n = 19) | ***P* value^b^** | **NAs** |
| *APOE* ε4, No. (%) | 3 (30) | 3 (16) | 0.63 | - |
| Female, No. (%) | 6 (60) | 12 (63) | 1 | - |
| Delay, d | 741 (172 - 783) | 631 (366 - 773) | 0.95 | - |
| Age, y | 72 (69 - 77) | 71 (66 - 73) | 0.43 | - |
| Education, y | 10 (8 - 11) | 9 (9 - 12) | 0.88 | 1 \| 4 |
| MMSE, /30 | 22 (21-25) | 28 (27-29) | **< 0.001** | 0 \| 3 |
| CSF, pg/ml |  |  |  |  |
| Aβ_42/40_ | 0.032 (0.027 - 0.049) | 0.068 (0.051 - 0.074) | 0.14 | 4 \| 11 |
| Aβ_42_ | 708 (588 - 749) | 738 (636 - 1180) | 0.46 | 1 \| 8 |
| p-tau_181_ | 71 (66 - 98) | 44 (34 - 71) | **0.031** | 0 \| 8 |
| t-tau | 549 (507 - 803) | 310 (240 - 450) | **0.018** | 0 \| 8 |
| Plasma, pg/ml |  |  |  |  |
| Simoa - Aβ_42/40_ | 0.055 (0.041 - 0.057) | 0.057 (0.052 - 0.062) | 0.26 | 2 \| 2 |
| IPMS-Shim - Aβ_42/40_ | 0.016 (0.015 - 0.017) | 0.018 (0.017 - 0.021) | **< 0.01** | - |
| IPMS-Shim - Composite | 5.1 (4.7 - 5.3) | 4 (2.7 - 4.7) | **< 0.01** | - |

^a^: 8 MCI, 5 SCI, 3 NDD and 3 OND; **^b^**: Fisher’s exact (*APOE* ε4 and sex) or Mann-Whitney *U* tests

**Fig. S1** Performance of the plasma amyloid biomarkers to discriminate A+T+ from A-T- subjects. ROC analysis of the amyloid biomarkers. AUC is presented with 95% confidence interval (CI).


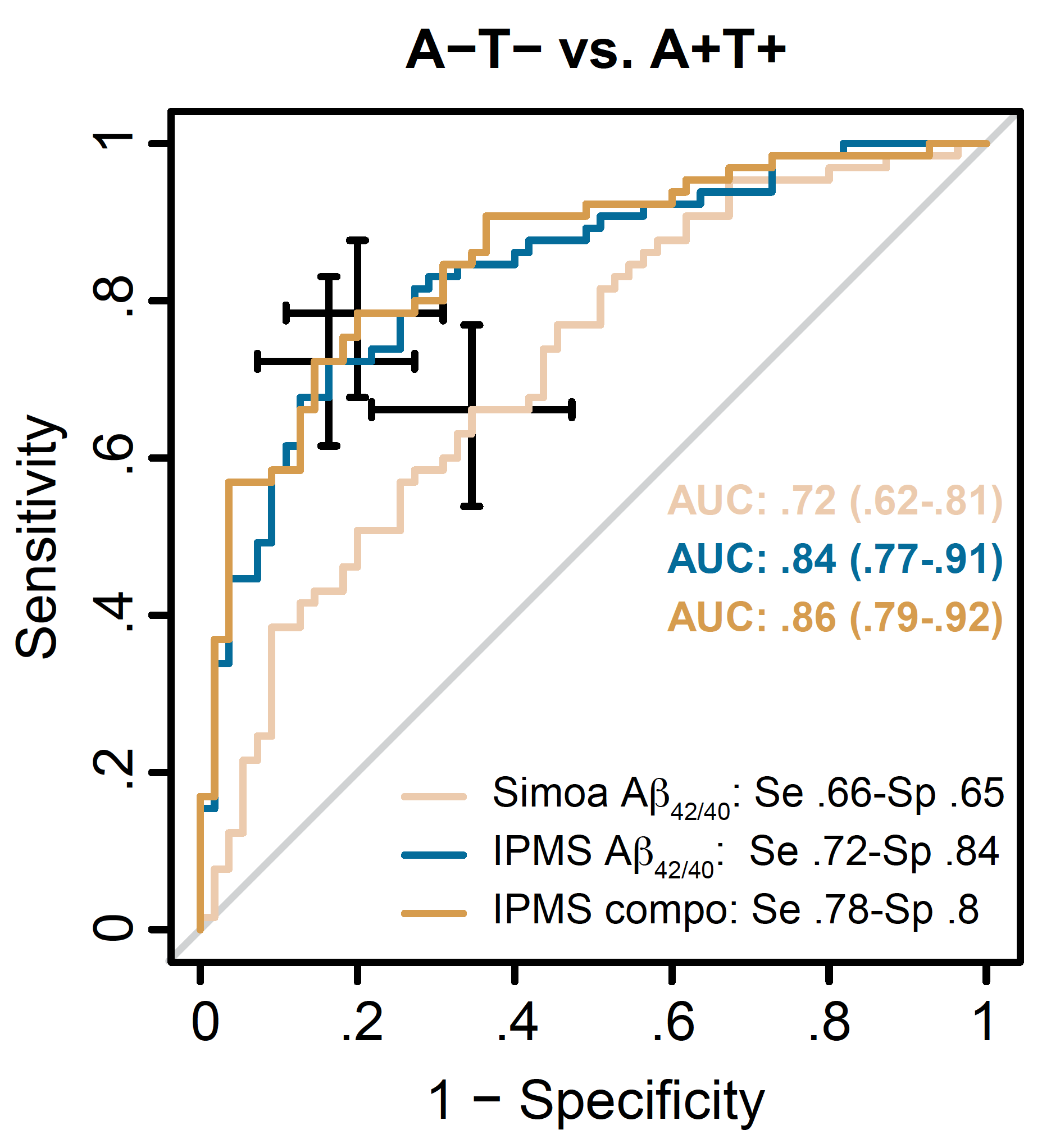

Supplement: Supplementary file 1 — Additional file 1: Table S1. Details about the diagnosis in the other neurodegenerative diseases (NDD) and other neurological disorders (OND) groups. Table S2. Correlations between plasma Biomarkers with Simoa and IPMS-Shim and Demographic Features. Table S3. Characteristics of Study Participants According to CSF Aβ42/40 Status and AT or AT(N) Profiles. Table S4. Correlations between Plasma Amyloid Biomarkers and CSF/Plasma Amyloid Biomarkers. Table S5. Baseline Characteristics of the 29 Individuals with follow-up and repeated biomarkers assessment. Fig. S1. Performance of the plasma amyloid biomarkers to discriminate A+T+ from A-T- subjects. ROC analysis of the amyloid biomarkers. AUC is presented with 95% confidence interval (CI). [file 13195_2023_1188_MOESM1_ESM.docx]
